# IRDC-Net: An Inception Network with a Residual Module and Dilated Convolution for Sign Language Recognition Based on Surface Electromyography

**DOI:** 10.3390/s23135775

**Published:** 2023-06-21

**Authors:** Xiangrui Wang, Lu Tang, Qibin Zheng, Xilin Yang, Zhiyuan Lu

**Affiliations:** 1 School of Health Science and Engineering, University of Shanghai for Science and Technology, Shanghai 200093, China; yepraywong@foxmail.com (X.W.); qbzheng@usst.edu.cn (Q.Z.); leah188800@gmail.com (X.Y.); 2 School of Rehabilitation Science and Engineering, University of Health and Rehabilitation Sciences, Qingdao 266072, China; zhiyuan.lu@uor.edu.cn

**Keywords:** sign language recognition, surface electromyogram, inception network, residual module, dilated convolution

## Abstract

Deaf and hearing-impaired people always face communication barriers. Non-invasive surface electromyography (sEMG) sensor-based sign language recognition (SLR) technology can help them to better integrate into social life. Since the traditional tandem convolutional neural network (CNN) structure used in most CNN-based studies inadequately captures the features of the input data, we propose a novel inception architecture with a residual module and dilated convolution (IRDC-net) to enlarge the receptive fields and enrich the feature maps, applying it to SLR tasks for the first time. This work first transformed the time domain signal into a time–frequency domain using discrete Fourier transformation. Second, an IRDC-net was constructed to recognize ten Chinese sign language signs. Third, the tandem CNN networks VGG-net and ResNet-18 were compared with our proposed parallel structure network, IRDC-net. Finally, the public dataset Ninapro DB1 was utilized to verify the generalization performance of the IRDC-net. The results showed that after transforming the time domain sEMG signal into the time–frequency domain, the classification accuracy (acc) increased from 84.29% to 91.70% when using the IRDC-net on our sign language dataset. Furthermore, for the time–frequency information of the public dataset Ninapro DB1, the classification accuracy reached 89.82%; this value is higher than that achieved in other recent studies. As such, our findings contribute to research into SLR tasks and to improving deaf and hearing-impaired people’s daily lives.

## 1. Introduction

Sign language, an auxiliary tool to help deaf and hearing-impaired people to communicate, is primarily conveyed through hand and arm gestures [1]. The latest estimations indicate that hearing impairments affect 1.59 billion people worldwide [2]. In order to help deaf people to better integrate into social life, sign language recognition (SLR), which is an important application of hand gesture recognition technologies, have been paid increased attention by researchers in recent years.

Hand movements are the result of the central nervous system driving different upper limb muscles. A surface electromyogram (sEMG), which reflects the intensity of the muscle contraction triggered by the motor unit action potential [3], can be recorded in a non-intrusive fashion using surface electrodes [4]. Multichannel sEMG signals, recorded using sEMG sensors placed on the arms, provide a wealth of information about the coactivation and coordination of multiple muscles that are associated with different sign gestures [5]. Therefore, sEMG-based methods provide researchers with a significant opportunity to decode the muscular activity involved in human hand gesture recognition and distinguish subtle finger configurations, hand shapes, and wrist movements [6]. Traditional sEMG-based SLR methods are generally based on machine learning (ML) algorithms that extract hand-crafted features from the raw sEMG signals as input to train the classifier [7,8,9]. As a consequence, offsetting the subjectivity of hand-crafted features and the uncertainty of feature subsets can improve the final classification accuracy.

With the aim of automatically extracting high-level features from the raw sEMG signals, SLR methods based on deep learning (DL) have attracted widespread attention from researchers. Convolutional neural networks (CNNs), as spatial characteristic extractors, are DL algorithms that utilize the convolution kernel parameters learned from gradient descent to perform convolution on the input data so as to obtain the high-level features of the input data [10].

The network structures of CNNs can be divided into two main categories: tandem structures and parallel structures. The tandem structure is the original form of a CNN; here, the semantic information of the input data is acquired from each stacked convolutional layer. Wang et al. [11] employed a one-dimensional (1-D) CNN with VGG-net [12] to recognize six different Chinese sign language signs. Li et al. [13] designed a CNN architecture with three convolutional layers to obtain high-level representations of the sEMG signal matrix; then, they used a bidirectional long short-term memory network (Bi-LSTM) as a temporal modelling layer to obtain the temporal characteristics. It follows that increasing the number of convolutional layers enhances the feature extraction capability of the tandem network. However, for sequences with long-term dependent information, such as sEMG signals, the tandem structure cannot adequately extract the features of the input data [14,15]. To address this problem and reduce computational costs, Szegedy et al. [16,17,18,19] proposed a parallel CNN architecture, called Inception. The Inception network fuses the outputs of each branch containing different convolutional kernel sizes to form a feature map with more satisfactory semantic information than the tandem architecture.

Although the Inception architecture enriches the feature map, it cannot enlarge the receptive field, which determines the scope of the network’s observation of the input data. A larger receptive field facilitates the network’s capability to capture contextual information, thus improving the accuracy of the model [20]. In recent years, the focus of CNN research has gradually shifted towards enlarging the receptive field. There are two commonly used methods for enlarging receptive fields: (1) deepening the networks, and (2) increasing the kernel size. According to the work of He [21], deepening the networks leads to degradation problems during the model training process. Increasing the kernel size leads to a corresponding increase in the network parameters, which makes the model more difficult to train [22]. He et al. [21] embedded a residual module into a CNN in computer vision tasks to address the degradation problem caused by deepening networks. Yu et al. [23] proposed a dilated convolution to enlarge the receptive field while avoiding the increase in the model parameters. Therefore, the residual module and the dilated convolution provide a promising basis for increasing the receptive field in the Inception architecture in one-dimensional signal recognition tasks.

Based on the above analysis and focusing on long-term sequence sEMG sign language signal recognition, we used the Inception architecture as the backbone of our classification framework to obtain sufficient feature information for the sEMG signal. Furthermore, to further improve the accuracy of the sEMG-based SLR tasks, we first combined the dilation convolution and residual modules to avoid the degradation problem while enlarging the receptive field. The main contributions and novelties of this study are as follows:

(1) One-dimensional discrete Fourier transformation was used to transform the non-stational time domain sEMG signal into a time–frequency domain, enhancing the characteristics of the time domain signal and further improving the accuracy of the SLR task.
(2) A novel Inception architecture with a residual module and dilated convolution (IRDC-net) was proposed in this study; it was applied to SLR tasks for the first time. The IRDC-net enriched the sEMG feature map and enlarged the receptive field while avoiding model degradation, meaning that it is suitable for sEMG classification tasks with long-term dependent information.
(3) The public dataset Ninapro DB1 [24] was used to test the generalization performance of our proposed model; the results showed that our methods led to better performance than other recent studies that utilized Ninapro DB1, indicating that IRDC-net can be applied to a wider range of SLR tasks.

## 2. Materials and Methods

**Figure 1:**
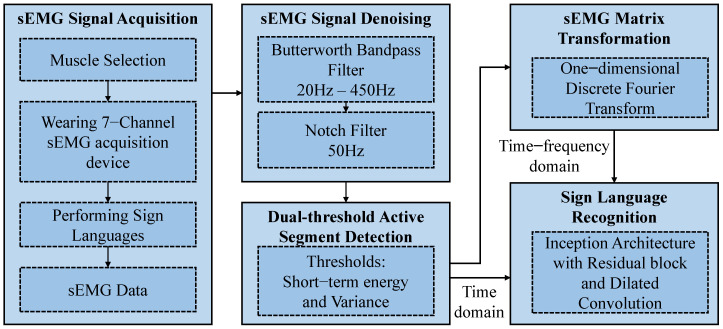
Block diagram of the proposed framework for hand gesture recognition.

Figure 1 shows a block diagram of the hand gesture recognition method using multichannel sEMG signals. The sEMG signal acquisition was carried out on a computer with an Inter(R) Xeon(R) Silver 4110 CPU @ 2.10 GHz and an RTX 2080 Ti GPU, with 11 GB RAM. The signal processing platform includes Matlab 2018b and a Python integrated development environment called Pycharm. Signal denoising and segmentation were based on Matlab 2018b, while the signal matrix transformation and deep learning model training and testing were based on Pycharm. IRDC-net was built based on a Python library, Keras 2.4.3.

### 2.1. sEMG Signal Acquisition

**Figure 2:**
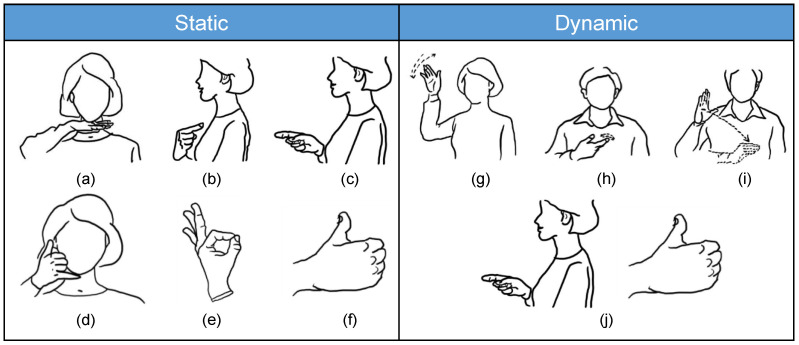
The ten Chinese sign language signs considered in this study. (**a**) Wait (等), (**b**) I (我), (**c**) you (你), (**d**) ring up (打电话), (**e**) OK (好的), (**f**) good (好), (**g**) goodbye (再见), (**h**) have (有), (**i**) morning (早上), (**j**) hello (你好). Note: this sign for “have” refers to somebody owning something.

Ten sign languages signs incorporating both static and dynamic gestures were employed in this study (Figure 2). Static sign language is defined as the finger, wrist, and upper limb maintaining just one form during the performance of sign language, while dynamic sign language is defined as the finger, wrist, and upper limb needing to adopt multiple forms during the performance of sign language.

**Figure 3:**
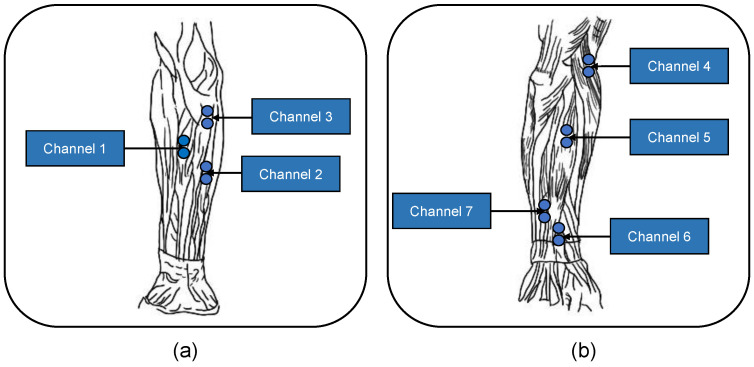
Placement positions of the electrodes. (**a**) The anterior group of antebrachial muscles and (**b**) the posterior group of antebrachial muscles. Blue solid circles denote the two Ag/AgCl electrodes corresponding to one channel.

The sEMG signal measurements were conducted with our self-made 1000 Hz-sampling rate sensor system. In each sEMG sensor, there are two Ag/AgCl electrodes with a contact dimension 15 mm in diameter and 10 mm electrode-to-electrode spacing. Seven channel sEMG sensors were located over seven sites on the surface of the forearm muscles: the flexor carpi radialis, the flexor digitorum superficialis, the palmaris longus, the brachioradialis, the extensor digitorum, the extensor pollicis longus, and the extensor digiti minimi. The 7 muscles correspond to channels 1–7 shown in Figure 3.

A group of 10 right-handed healthy subjects (age 24.8 ± 3.12), made up of 7 men and 3 women, participated in the data collection experiments; all participants were informed of the specific risks and benefits associated with the study. Before placing the electrodes, the skin of each subject’s upper limbs needed to be disinfected with 70% medical alcohol to ensure that the skin was in a dry state. Each subject was required to practice the sign language movements until muscle memory was formed prior to signal acquisition.

During signal acquisition, each subject performed the 10 selected Chinese sign language signs in a sequence, with 5 repetitions per motion. The 5 repetitions were separated by 4 s intervals. Each subject took a 2 min break after a movement cycle to avoid muscle fatigue. When the subject finished performing the sign language, their resting posture involved sitting in a chair with a straight back and upper limbs lying flat on the table. Each subject performed a total of 50 sign language movements (10 sign language gestures repeated 5 times).

### 2.2. sEMG Signal Pre-Processing and Segmentation

The raw sEMG signal contains various noises caused by motion artifacts and power frequency interference [25]. Thus, we first utilized a 20–450 Hz Butterworth bandpass filter to filter out noise derived from motion artifacts. Then, a 50 Hz notch filter was used to filter the power frequency interference noise.

**Figure 4:**
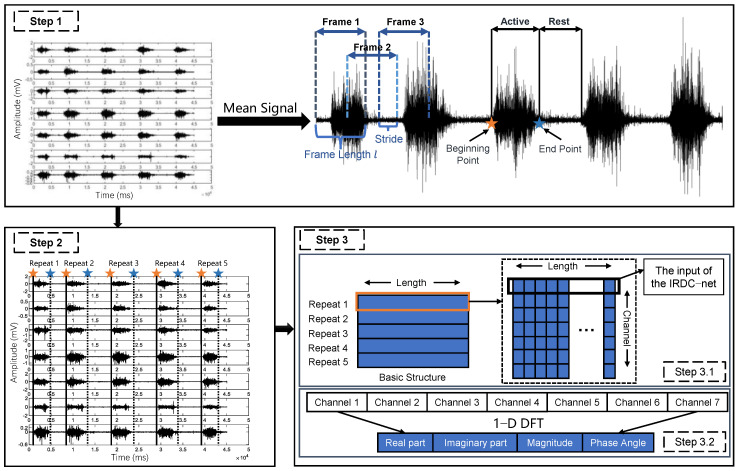
Illustration of data segmentation. Step 1 and Step 2 describe the 7−channel sEMG signal segmentation process of one sign language movement, Step 3 describes the process of constructing the time domain (Step 3.1) and the time−frequency domain (Step 3.2) datasets. The orange box in Step 3.1 represents the seven−channel sEMG signal of Repeat 1.

In the active segment detection method, the short-term energy (*E*) and variance (*Var*) were used as the threshold to detect the start and end points of the active segment. As shown in Step 1 of Figure 4, we first calculated the average of the 7-channel sEMG signal to obtain the single-channel mean signal of the original 7-channel signal and used a sliding window to frame the mean signal. Then, we calculated the *E* and *Var* of each frame; finally, we took a frame larger than the threshold as the start point of the active segment and a frame smaller than the threshold as the end point of the active segment. The two thresholds were defined as

(1) $$E=\frac{1}{l}\overset{l}{\underset{n=0}{\sum }}x{\left(n\right)}^{2},$$

(2) $$Var=\frac{1}{l}\sum_{n=0}^{l}{\left(x\left(n\right)-x{\left(n\right)}_{avg}\right)}^{2},$$

where l denotes the length of the sliding window, namely, the frame length, x(n) is one of the elements of a frame, and $x{\left(n\right)}_{avg}$ is the average value of all of the elements in a frame.

After calculating the start and end points of the active segment in the mean signal, as shown in Step 2 of Figure 4, we mapped the 2 points back to the original 7-channel signal to intercept the 5 active segments of the 7-channel signal. Since the length of the active segment of each repeat was different, we employed linear interpolation to make the length of each active segment equal to the maximal active segment length, i.e., 2664 ms.

### 2.3. Signal Matrix Transformation

To obtain the time–frequency domain information of the 7-channel sEMG signal, we employed sliding-window-based 1D discrete Fourier transformation (DFT). First, we split the time domain signal into frames using a sliding window (frame length: 20 ms, 50 ms, and 100 ms; overlap: 40% of the frame length) and performed 1D DFT on each frame separately. The definition of 1D DFT is shown as Equation (2). x(n) is the corresponding amplitude of each channel sEMG signal, where n is the index of the sEMG signal and N is the length of the discrete time domain signal; k denotes the index of the frequency domain signal. According to Euler’s formula, Equation (2) can be transformed into Equation (3):

(3) $$X\left(k\right)=\overset{N-1}{\underset{n=0}{\sum }}x\left(n\right)\cdot e^{-\frac{j2\pi nk}{N}},$$

(4) $$X\left(k\right)=\sum_{n=0}^{N-1}x\left(n\right)\left[\cos\left(\frac{2\pi nk}{N}\right)-j\cdot \sin\left(\frac{2\pi nk}{N}\right)\right].$$

The time domain sEMG signals constitute the superposition of multiple frequency domain signals, which can be considered the components of the time domain signal. Using Equation (4), the real part, imaginary part, magnitude, and phase angle can be obtained, as shown in Equations (5)–(8):

(5) $$x_{real}\left(k\right)=\sum_{n=0}^{N-1}x\left(n\right)\left[\cos\left(\frac{2\pi nk}{N}\right)\right],$$

(6) $$x_{imag}\left(k\right)=\overset{N-1}{\underset{n=0}{\sum }}-\left\{x\left(n\right)\left[\sin\left(\frac{2\pi nk}{N}\right)\right]\right\},$$

(7) $$x_{mag}\left(k\right)=\sqrt{x_{real}{\left(k\right)}^{2}+x_{imag}{\left(k\right)}^{2}},$$

(8) $$x_{\emptyset }\left(k\right)=tan^{-1}\left[\frac{x_{imag}\left(k\right)}{x_{real}\left(k\right)}\right].$$

Then, the 7-channel time domain sEMG signal matrix can be transformed into a matrix of frequency domain signals over time, referred to as the time–frequency domain sEMG matrix, as shown in Step 3.2 of Figure 4.

### 2.4. Sign Language Recognition: IRDC-Net

**Figure 5:**
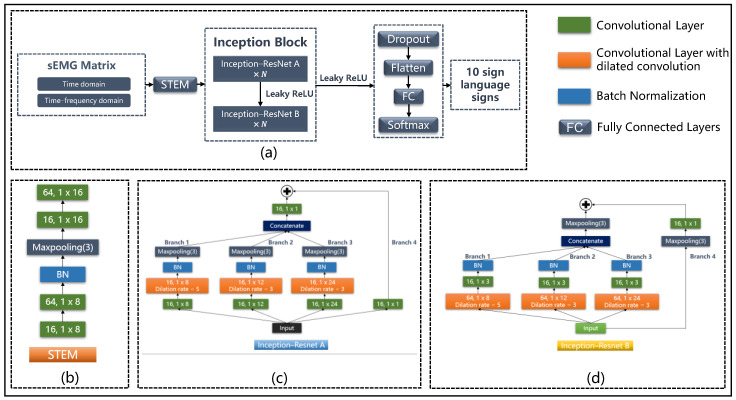
The framework of the proposed IRDC-net. (**a**) IRDC-net framework and (**b**) STEM block, and (**c**) the Inception-ResNet A block and (**d**) the Inception-ResNet B block. The form of the convolutional layer was “number of filters, kernel sizes”.

The main goal of IRDC-net was to enlarge the receptive field and enrich the spatial-temporal characteristics extracted by the convolutional layers. The framework of IRDC-net is shown in Figure 5. The network architecture mainly consists of a STEM block, an Inception block, a dropout layer, a flattening layer, a fully connected layer, and, finally, the softmax activation function. The STEM block is the first part of the Inception-related model [16,17,18,19]; it usually consists of a number of convolution layers and pooling layers. The convolution layer was used to extract low-level features and prepare for the subsequent Inception block’s extraction of higher-level features. Meanwhile, the pooling layer was used to reduce the dimensionality of the feature maps. We first used the STEM block (Figure 5b) to reduce the dimensionality in order to reduce computation and obtain preliminary features. Then, the Inception block was used to enrich the feature information contained in the input data. For the rest of the IRDC-net, we set the dropout rate to 0.8 for the following dropout layer. Since the activation function of leaky rectified linear units (Leaky ReLU) [26] can prevent the vanishing gradient problem and the dead neuron problem caused by the activation function ReLU [27], the Leaky ReLU function was used as the activation function of our proposed model.

#### 2.4.1. Receptive Field

**Figure 6:**
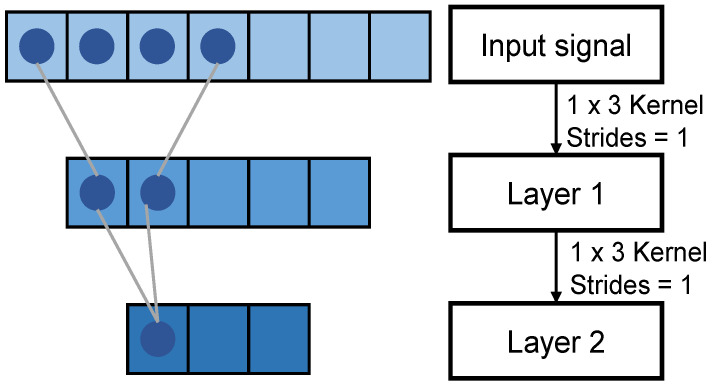
Representation of the receptive field.

The feature map extracted by the convolutional layers can be mapped back to the input signal; the size of the feature map mapped back to the input is the receptive field size. As shown in Figure 6, after the 2 convolutional filters with kernel sizes of 1 × 3 and 1 × 2, each element in Layer 2 contains information about the input signal with a length of 4. The larger the size of the receptive field, the more information the feature map contains about the input signal, which means the feature map may contain more global and higher-level semantic features. Hence, a large receptive field is suitable for long-term sEMG-signal-based SLR tasks.

#### 2.4.2. Inception Block

The large receptive field captures global information from the input data, but it also loses the local information of the input. To address this problem, in the Inception-ResNet A module (Figure 5c), kernel sizes of 1 × 8, 1 × 12, and 1 × 24 were used in Branch 1, Branch 2, and Branch 3, respectively. Moreover, the receptive field sizes of the next layer relative to the previous layer are 1 × 8, 1 × 12, and 1 × 24, which means that each element of the feature map contains information about the previous layer with length of 8, 12, and 24, respectively. In the Inception-ResNet B module (Figure 5d), we used 64 convolutional kernels with sizes of 1 × 8, 1 × 12, and 1 × 24 for Branch 1, Branch 2, and Branch 3, respectively, in order to obtain 3 feature maps with different characteristic information; then, 16 convolutional kernels with a size of 1 × 3 were used to reduce the dimensionality. Branch 4 of Inception-ResNet A and Inception-ResNet B was the residual module. The residual module in Branch 4 and the dilated convolution modules in Branch 1 to Branch 3 are described in Section 2.4.3 and Section 2.4.4.

#### 2.4.3. Residual Module

**Figure 7:**
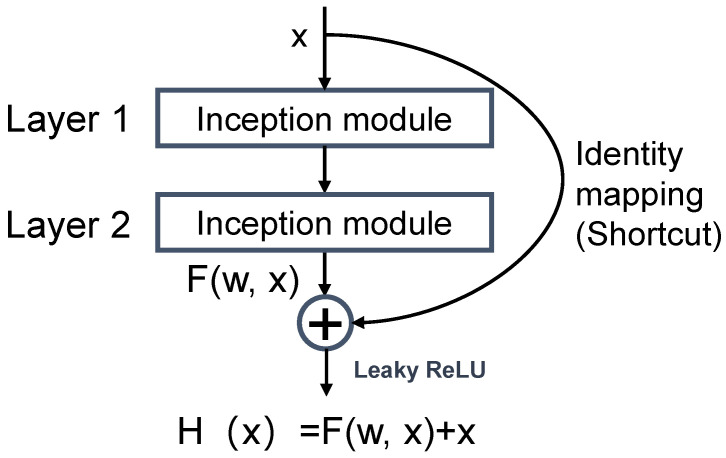
Residual block of IRDC-net.

The residual module introduced the identity mapping property to prevent model degradation. Assuming that Layer 1 and Layer 2 (Figure 7) were the deep layers of a network, in order to prevent degradation, the output *H*(*x*) must be equal to the input *x*, which can be described as *H*(*x*) = *x*; this is referred to as identity mapping. Through the identity mapping, the effects of Layer 1 and Layer 2 in the network can be eliminated, so we can deepen the network by using a shortcut to connect every two or three convolution layers without considering degradation.

Conventional CNN has difficulty fitting the identity mapping function H(x)=x, so a residual module was designed for the identity mapping function:

(9) H(x)=F(w, x)+x,

where w is the weight of the previous layers. If F(w, x)=0, the identity mapping function H(x)=x can be constructed. Hence, F(w, x) becomes the residual that should be learned.

#### 2.4.4. 1D Dilated Convolution

**Figure 8:**
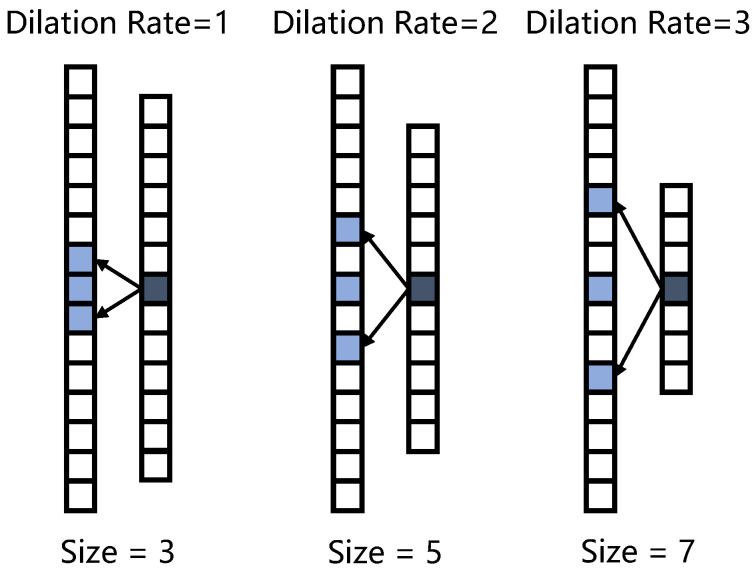
Representation of dilated convolution. The “size” at the bottom indicates the receptive field size. The blue box denotes the convolution kernel. The blue-grey box denotes the convolution results. A dilation rate of 1 means that the convolution kernel was still a standard kernel.

Unlike a standard convolution kernel, each element of a dilated convolution kernel has a space, and the space length is called the dilation rate. As shown in Figure 8, the receptive field size increased after using dilated convolution. Note that the parameters produced by a convolutional layer are as follows: output channel×(kernel size×input channel+bias), where the input and output channels are equal to the number of the convolution filters set at each dilated convolution layer. Dilated convolution does not cause an increase in parameters while expanding the receptive field because the kernel size does not increase. In this study, the dilation rate was set to 5 and 3 in the different branches of IRDC-net (Figure 5c,d).

## 3. Experiments and Results

### 3.1. Datasets

Mydata: This study recruited 10 subjects to acquire the sEMG signals of 10 sign language gestures. Therefore, the complete dataset was constructed from 10 × 10 = 100 basic structures (Step 3.1 of Figure 4). Since each sign language gesture was repeated five times and each row of the basic structure contains seven sEMG samples, the complete dataset contains a total of 100 × 5 × 7 = 3500 sEMG samples, and these 3500 sEMG samples were used as the input of IRDC-net.

Ninapro DB1: Moreover, we validated the effectiveness of the proposed IRDC-net using a public dataset, Ninapro DB1 [24]. Ninapro DB1 contains the upper limb sEMG signals of 27 subjects; each subject performed 52 gestures including finger, hand, and wrist movements. The sEMG data were acquired using Delsys Trigno with 10 Otto Bock MyoBock 13E200 electrodes with a sampling rate of 100 Hz.

For both Mydata and Ninapro DB1, the ratio of the training set to the test set was seven to three, and 30% of the training set was separated and used as the validation set. Stratified sampling was employed to split the dataset so that the proportion of each label in the training set and the test set was the same as in the original dataset.

### 3.2. Classification Performance with Different DFT Frame Lengths

This experiment aimed to find the optimal DFT frame length for the classification tasks. In the Inception block (Figure 5a), we set two Inception-Resnet A modules and one Inception-Resnet B module. For the time–frequency domain data, we performed 1D-DFT on the data with three different frame lengths (20 ms, 50 ms, and 100 ms) to obtain the frequency domain information of sEMG, which changed with time, for these different frame lengths.

In this experiment, the classification accuracy of IRDC-net reached 91.70% when the frame length was set to 50 ms. The classification accuracy of IRDC-net with frame lengths of 20 ms (81.40%) and 100 ms (90.87%) was lower than the classification accuracy at 50 ms. As shown in Table 1, the precision, recall, and f1 score of the 50 ms frame length also represented the best results. Therefore, a balance between temporal precision and the time domain data information capacity can be reached when the frame length is 50 ms.

### 3.3. Comparison of the Tandem Network Structure and the Parallel Structure

This experiment compared the classification performance between the parallel network IRDC-net and the tandem networks VGG-net [12] and ResNet-18 [21] using Mydata. The frame length of the DFT was set to 50 ms. The hyper-parameter settings are shown in Table A1 and Table A2 (Appendix A). As shown in Table 2, the classification accuracies of the parallel IRDC-net (time domain, 84.29%; time–frequency domain, 91.70%) were all higher than those of the tandem VGG-net (time domain, 31.43%; time–frequency domain, 82.42%) and ResNet-18 (time domain, 60.76%; and time–frequency domain, 83.43%), which demonstrated that the parallel network structure performed better than the tandem structure.

### 3.4. Comparison of the Inception-Related Networks and IRDC-Net

**Figure A1:**
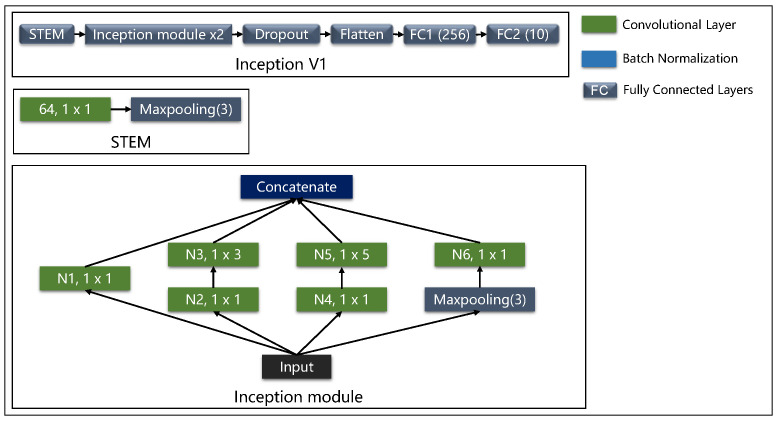
The Inception-V1-based model. We stacked two Inception modules, and N1–N6 were the number of convolution filters. For the first Inception module, N1–N6 were set to [64, 96, 128, 16, 32, and 32], respectively; for the second Inception module, N1–N6 were set to [128, 128, 192, 32, 96, and 64], respectively.

**Figure A2:**
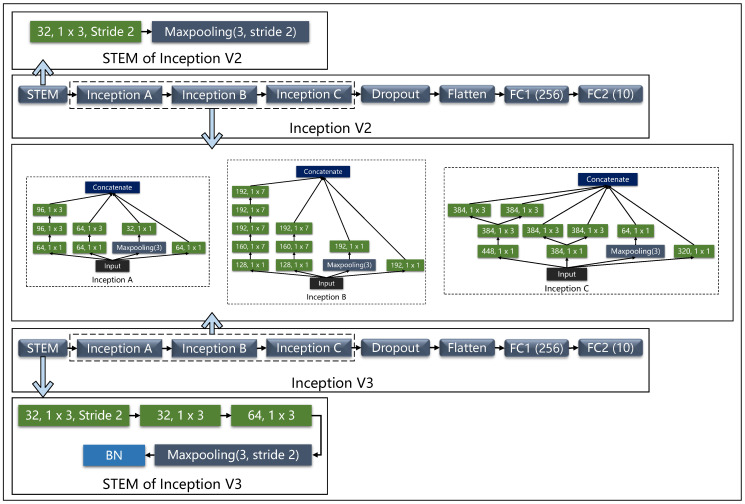
The Inception-V2- and V3-based models. The two model share the architecture of Inception A, Inception B, and Inception C. The colour of each module is consistent with Figure A1.

Since the proposed IRDC-net was an improved version of the Inception network, we justified improving the model by using three basic Inception-related networks that did not use dilated convolution and residual modules, namely, the Inception-V1-based, Inception-V2-based, and Inception-V3-based models. The model architecture is presented in Figure A1 and Figure A2 (Appendix A) and the comparison results are shown in Table 3. The hyper-parameter settings are shown in Table A1 and Table A2 (Appendix A).

### 3.5. Results of the Public Dataset Ninapro DB1

In order to evaluate the rationality and effectiveness of our proposed network architecture, we also tested its classification performance on a public dataset, Ninapro DB1. Since the Ninapro DB1 dataset contains the sEMG signals of 52 upper limb gestures, requiring a model with strong feature capture capabilities, the Inception block increased from two Inception-ResNet A modules and one Inception-ResNet B module for Mydata to six Inception-ResNet A modules and two Inception-ResNet B modules (Figure 5a). The hyper-parameter settings are shown in Table A3 (Appendix A).

As noted above, the time–frequency domain data contain both temporal and spatial information; therefore, we also employed 1D-DFT to obtain the time–frequency domain data for Ninapro DB1, and the frame length was set to 500 ms. Table 4 shows the classification results for Ninapro DB1 using our proposed IRDC-net and the comparative results of other studies based on Ninapro DB1. When classifying 52 different movements, our proposed method achieved accuracy levels of 89.82%, exhibiting better performance than other CNN-based methods.

### 3.6. Ablation Experiments

**Figure 9:**
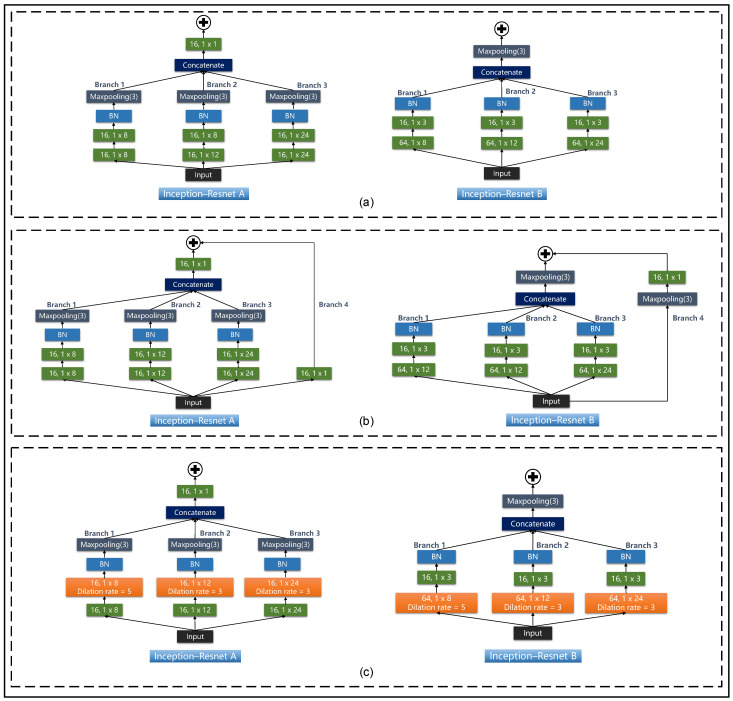
Model structures for the ablation experiments. (**a**–**c**) show Experiment 1, Experiment 2, and Experient 3 of Table 5, respectively. The colour of each module is consistent with Figure 5.

The main goal of IRDC-net was to increase the receptive field to adapt to the task of classifying long-term sequences and to enhance the feature capture capabilities of long-term sequences. Therefore, this study combined dilated convolution and a residual module to expand the receptive field while avoiding the model degradation problem caused by increasing the depth of the model. These ablation experiments verified that the decrease in classification performance due to the reduced receptive field size was caused by (a) the removal of the dilated convolution module and (b) the model degradation problem after the removal of the residual module. The ablation experiments were all based on the Mydata and Ninapro DB1 datasets, and the time–frequency information for the two datasets was used as the input of the classification model. The model structures of this ablation experiment are shown in Figure 9.

As shown in Table 5, Experiment 1 used IRDC-net without the residual module and dilated convolution as a baseline model. The accuracy levels exhibited in Experiment 2 and Experiment 3 for Mydata and Ninapro DB1 were significantly higher than those in Experiment 1. The results of the ablation experiments verified the effectiveness of dilated convolution and the residual module.

To further verify the effect of dilated convolution, the dilation rate in every branch of the IRDC-net (Figure 5b,c) was set to 1, which converted the dilated convolution to standard convolution. As shown by Experiment 2 (Table 5), after removing the dilated convolution, the accuracy for Mydata and Ninapro DB1 decreased from 91.70% and 89.82% to 87.01% and 87.83%, respectively. This result demonstrates that reducing the receptive field size will reduce the classification performance.

To verify the ability of the residual module to solve model degradation, we removed Branch 4 of the Inception block of IRDC-net (Figure 5b,c) to test whether model degradation would occur. Experiment 3 (Table 5) shows that after eliminating the residual module, the classification accuracy decreased from 91.70% and 89.82% to 83.17% and 63.07% for Mydata and Ninapro DB1, respectively. The results of Experiment 2 and Experiment 3 also demonstrate that the residual module plays a more important role in the proposed IRDC-net than the dilated convolution.

## 4. Discussion

This study first demonstrated that the classification performance of parallel structure networks, such as the proposed IRDC-net, is better than that of tandem networks such as VGG-net and ResNet-18 (Table 2). The proposed IRDC-net summed the feature map output by the parallel convolutional kernel with different kernel sizes to construct a feature map with more comprehensive semantic information so as to improve the feature-capturing capability of model. Table 2 also shows that after transforming the time domain data into a time–frequency domain to acquire data that represent not only the time domain information but also the time–frequency domain, the classification accuracy increased by 7.41% for Mydata when using IRDC-net. The accuracy increased because the sEMG is non-stationary and non-linear, and the physiological characteristics of the time domain signal are not obvious. Since the time domain signal constitutes the superposition of multiple sub-signals, the frequency domain information of the sub-signals can be calculated using 1D DFT. Moreover, by using the calculation results of 1D DFT as the input of the IRDC-net, the characteristics of the time domain signal are enhanced, so the classification model can learn the characteristics of the input more easily.

**Figure 10:**
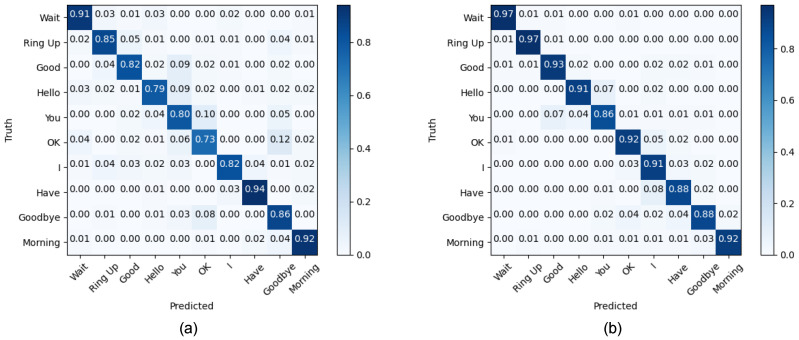
Confusion matrix of IRDC-net for Mydata. (**a**) The results of the time domain signal and (**b**) of the time–frequency domain signal.

As shown in Figure 10, we also presented a confusion matrix for the classification results of IRDC-net on Mydata. For the time domain dataset of Mydata, the recall of “OK” was relatively poor. After applying DFT, the recall increased from 0.73 to 0.92. This result almost concurs with the discussions in the previous paragraph. However, for the dynamic sign language gesture “Have”, the recall decreased from 0.94 to 0.88. The reason for this may be that the degree of muscle activation during dynamic gestures varied greatly among subjects; therefore, some uncertainty about the recognition results was still present. Figure 10 also shows that there was a mutual misclassification between “Hello”, “You”, and “Good”, since the dynamic sign language gesture for “Hello” consists of the gestures for “You” and “Good”, and the distance between the features may be smaller than it is for other highly differentiated sign language gestures. There is a chance that when the subjects performed “Hello”, the gesture for “You” took longer to perform than the gesture for “Good”, causing the whole signal to be biased towards “You”. Therefore, the dynamic sign language gesture for “Hello” was misclassified as “You” and “Good”.

**Figure 11:**
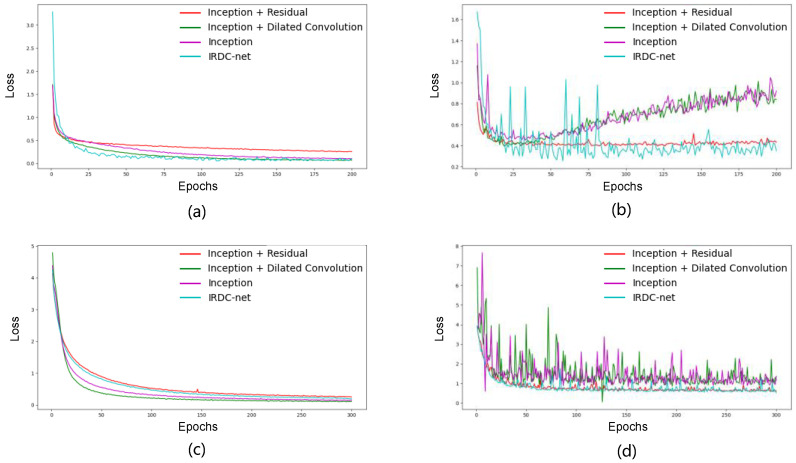
Loss curve for the ablation experiments. (**a**,**c**) Training curves of Mydata and Ninapro DB1, respectively; (**b**,**d**) validation curves of Mydata and Ninapro DB1, respectively.

The results also demonstrated that the dilated convolution and residual module improve the classification performance (Table 5). Figure 11b,d show that, after removing the dilated convolution, the training error increased slightly. This is because dilated convolution works to enlarge the receptive field size, which can then capture more information from the model’s input; the increase in the training error can, therefore, be attributed to the reduction in the receptive field size.

For the residual module, in order to enlarge the receptive field size, we not only employed dilated convolution but also increased the depth of the network. However, deepening the network can cause degradation problems. The residual module has the function of identity mapping, which makes the output of a convolutional layer equal to the input. When using a shortcut to connect two or three convolutional layers (e.g., Branch 4 in Figure 5c,d) to transmit information from the shallower network to the deeper network, the accuracy of the deeper network does not decrease due to the optimal shallower network. As shown in Figure 11, the loss value of IRDC-net without a residual module (the purple line and the green line) increased after 50 epochs for Mydata (Figure 11b); this demonstrates that the model not only degraded but also overfitted without the residual module. Meanwhile, the loss value fluctuated significantly for Ninapro DB1 (the purple line and the green line in Figure 11d). We verified the anti-degradation function of the residual module on two datasets, and the results showed that increasing the model’s depth caused model degradation problems, which reduced the fitting ability of the model.

## 5. Conclusions

This study proposed a novel network based on the Inception architecture with a residual module and dilated convolution (IRDC-net). It was used to recognize 10 Chinese sign language gestures, and we used the public dataset Ninapro DB1 to verify the effectiveness of the proposed IRDC-net. Then, 1-D DFT was employed to transform the time domain information into time–frequency domain information. The results showed that the classification accuracy for Mydata increased by 7.41% after using 1D DFT. When using the time–frequency information from Mydata as the input for IRDC-net, the classification accuracy reached 91.70%, which was higher than that of the tandem VGG-net and ResNet-18. This result indicates that the proposed parallel IRDC-net not only enhanced the feature capture capability of the classification model but also avoided the model degradation problem; it exhibited improved accuracy for sEMG-based SLR tasks and was superior to the traditional CNN method.

Although the proposed IRDC-net achieved better results in the sEMG-based SLR task, it still has some limitations. The number of participants plays a crucial role in model generalization. This study recruited 10 participants for the sEMG signal acquisition. Future work will recruit more participants to make the SLR study more reasonable and generalizable. Additionally, this work only used sEMG as the model input. However, at present, more and more research works focus on using a combination of sEMG and kinematics parameters as the model input so that the model not only acquires the bio-information of sign language gestures but also learns users’ spatial motion information. In addition, the dynamic gestures and some highly similar sign language gestures led to misclassification problems. Future work will focus on the design of a multimodal classification framework that fuses the sEMG signal with kinematics information. In addition, we will explore a metric learning method to narrow down the features of the same sign language gestures in order to better discriminate dynamic and similar gestures.

## Figures and Tables

**Table 1:** Evaluation metric results for different DFT frame lengths.

Frame Length	25 ms			50 ms			100 ms		
Metrics	Precision	Recall	f1 Score	Precision	Recall	f1 Score	Precision	Recall	f1 Score
class 0	91.27%	94.06%	92.13%	96.52%	97.68%	97.61%	97.33%	98.27%	97.30%
class 1	98.14%	96.25%	97.21%	97.68%	97.68%	97.68%	100.00%	98.34%	99.13%
class 2	83.64%	85.78%	84.52%	91.49%	93.02%	92.26%	93.73%	92.51%	93.66%
class 3	81.87%	75.60%	78.74%	95.48%	91.50%	93.52%	92.66%	94.97%	93.82%
class 4	65.53%	66.32%	66.85%	87.49%	86.78%	86.13%	83.68%	86.90%	84.14%
class 5	83.28%	83.28%	83.28%	90.07%	92.46%	91.25%	87.62%	83.57%	85.12%
class 6	81.77%	86.54%	83.67%	83.14%	91.51%	87.65%	88.61%	89.46%	88.54%
class 7	78.22%	80.18%	79.43%	92.70%	88.44%	90.57%	85.79%	87.27%	86.55%
class 8	89.30%	81.47%	85.39%	87.82%	88.50%	87.67%	91.19%	88.24%	89.43%
class 9	91.72%	90.03%	91.%	99.38%	92.47%	95.42%	92.50%	91.42%	91.46%
Average	84.47%	83.95%	84.22%	**92.18%**	**92.00%**	**91.98%**	91.31%	91.10%	90.92%

Bold indicates the best results.

**Table 2:** Comparison of the classification performance of the parallel network IRDC-net and the tandem networks VGG-net and ResNet-18.

	Accuracy	
Model	Time Domain	Time–Frequency Domain
IRDC-net	**84.29%**	**91.70%**
VGG-net	67.33%	84.29%
ResNet-18	70.85%	83.84%

Bold indicates the best results.

**Table 3:** Comparison of the classification performance of the Inception-related networks.

	Accuracy	
Model	Time Domain	Time–Frequency Domain
IRDC-net	**84.29%**	**91.70%**
Inception-V1-based model	70.00%	86.81%
Inception-V2-based model	78.29%	87.11%
Inception-V3-based model	78.95%	87.58%

Bold indicates the best results.

**Table 4:** Comparison of the classification accuracies for the public dataset Ninapro DB1.

Years	CNN Models	Accuracy
2020 [28]	A CNN model with an evolutionary algorithm	81.4 ± 4.0%
2021 [29]	Hierarchical-view pooling network	88.4%
2022 [30]	Deformable convolutional network	83.10%
2022 [31]	Dual-view multiscale convolutional network	86.72%
2022 [32]	Concatenate feature fusion recurrent convolutional network	88.87%
2022 [33]	Multi-stream convolutional block attention module–gate recurrent unit	89.70%
Ours	IRDC-net	**89.82%**

Bold indicates the best results.

**Table 5:** Results of ablation experiments on Mydata and Ninapro DB1.

		Accuracy	
	Method	Mydata	Ninapro DB1
Experiment 1	Inception module (Figure 9a)	81.67%	75.99%
Experiment 2	Inception module + residual module (Figure 9b)	87.01%	87.83%
Experiment 3	Inception module + dilated convolution (Figure 9c)	83.17%	80.55%
Experiment 4	IRDC-net (Figure 5)	**91.70%**	**89.82%**

Bold indicates the best results.

## Data Availability

The self-collected dataset presented in this study are available on request from the corresponding author. The data are not publicly available due to the privacy of each subject’s bio-information and the future studies that should be carried out on the self-collected dataset. The public dataset Ninapro DB1 can be found here: http://ninapro.hevs.ch/node/2.

## Appendix A

**Table A1:** Hyper-parameter settings of the time domain signal for Mydata.

Hyper-Parameter	IRDC-Net	VGG-Net	ResNet-18	Inception V1-Based	Inception V2-Based	Inception V3-Based
Batch size	16	64	128	16	32	32
Epoch	200	100	100	100	100	100
Optimizer	Adam	Adam	Adam	Adam	Adam	Adam
Dropout rate	0.8	0.8	0.8	0.8	0.8	0.8
λ of l1 regularization	0.0001	0.0001	0.0001	0.0001	0.0001	0.0001
λ of l2 regularization	0.0001	0.0001	0.0001	0.0001	0.0001	0.0001

**Table A2:** Hyper-parameter settings of the time domain signal for Mydata.

Hyper-Parameter	IRDC-Net	VGG-Net	ResNet-18	Inception V1-Based	Inception V2-Based	Inception V3-Based
Batch size	16	16	128	16	32	32
Epoch	200	100	100	100	100	100
Optimizer	Adam	Adam	Adam	Adam	Adam	Adam
Dropout rate	0.8	0.8	0.8	0.8	0.8	0.8
λ of l1 regularization	0.0001	0.0001	0.0001	0.0001	0.0001	0.0001
λ of l2 regularization	0.0001	0.0001	0.0001	0.0001	0.0001	0.0001

**Table A3:** Optimal hyper-parameters for Ninapro DB1.

Hyper-Parameter	Value
Batch size	64
Epoch	300
Optimizer	Adam
Dropout rate	0.8
λ of l1 regularization	0.0001
λ of l2 regularization	0.0001
